# Early Experience with Tarlatamab (T-Cell Engagers) for Extensive-Stage Small Cell Lung Cancer (ES-SCLC) in Canada: Lessons Learned and Implementation Strategies

**DOI:** 10.3390/curroncol33020084

**Published:** 2026-01-31

**Authors:** Parneet K. Cheema, Kirstin A. Perdrizet, Randeep S. Sangha, Daniel Breadner, Nathalie Daaboul, Shannon Farley, Kevin Jao, Geoffrey Liu, Becky Logan, Barbara Melosky, Anthony Reiman, Stephanie Snow, Sunil Yadav, Shaqil Kassam

**Affiliations:** 1 Division of Medical Oncology, William Osler Health System, Brampton, ON L6R 3J7, Canada; kirstin.perdrizet@williamoslerhs.ca (K.A.P.); shannon.farley@williamoslerhs.ca (S.F.); 2 Faculty of Medicine, University of Toronto, Toronto, ON M5S 1A8, Canada; 3 Department of Oncology, Faculty of Medicine & Dentistry, University of Alberta, Edmonton, AB T6G 2R3, Canada; randeep.sangha@albertahealthservices.ca; 4 Division of Medical Oncology, Department of Oncology, Schulich School of Medicine & Dentistry, Western University, London, ON N6A 3K7, Canada; daniel.breadner@lhsc.on.ca; 5 Centre Intégré de Cancérologie de la Montérégie, Université de Sherbrooke, Longueuil, QC J4V 2H1, Canada; nathalie.daaboul@usherbrooke.ca; 6 Division of Medical Oncology and Hematology, Hôpital du Sacré-Coeur de Montréal, Montreal, QC H4J 1C5, Canada; kevin.jao@mail.mcgill.ca; 7 Division of Medical Oncology and Hematology, Department of Medicine, Princess Margaret Cancer Centre/University Health Network, University of Toronto, Toronto, ON M5G 2C4, Canada; geoffrey.liu@uhn.ca; 8 College of Medicine, Saskatchewan Cancer Agency, University of Saskatchewan, Saskatoon, SK S7N 4H4, Canada; becky.logan@saskcancer.ca (B.L.); sunil.yadav@saskcancer.ca (S.Y.); 9 Department of Medical Oncology, BC Cancer, Vancouver, BC V5Z 4E6, Canada; bmelosky@bccancer.bc.ca; 10 Department of Oncology, Saint John Regional Hospital, Saint John, NB E2L 4L2, Canada; anthony.reiman@horizonnb.ca; 11 Department of Medicine, Dalhousie University, Halifax, NS B3H 2Y9, Canada; 12 School of Integrated Health, University of New Brunswick, Fredericton, NB E3B 5A3, Canada; 13 Queen Elizabeth II Health Sciences Centre, Halifax, NS B3H 3A7, Canada; stephanie.snow@nshealth.ca; 14 Stronach Regional Cancer Centre, Southlake Health, Newmarket, ON L3Y 2P9, Canada; skassam@southlake.ca

**Keywords:** cytokine release syndrome, immune effector cell-associated neurotoxicity syndrome, bispecific antibody

## Abstract

As bispecific T-cell engagers (TCEs) gain traction in the oncology treatment landscape, cancer centres must develop robust clinical pathways to ensure their safe and efficient delivery. Given the limited experience of the Canadian medical oncology community with TCEs, collecting and publishing early clinical experiences with these novel agents will be essential to inform best practices and support their safe and effective adoption across the broader Canadian oncology community. The approval of tarlatamab, the first-in-class delta-like ligand 3 (DLL3)-targeted TCE for extensive-stage small cell lung cancer (ES-SCLC), underscores the importance of sharing early clinical experience with this agent, particularly given its unique safety profile, specific monitoring requirements, and use in a population that often has multiple comorbidities. Like other TCEs, tarlatamab is associated with cytokine release syndrome (CRS) and immune effector cell-associated neurotoxicity syndrome (ICANS), adverse events (AEs) that necessitate the development of dedicated protocols by medical oncologists and multidisciplinary inpatient and outpatient clinical teams to ensure prompt recognition and management of these associated toxicities. By sharing insights into administration protocols, dose ramp-up procedures, post-cycle 1 monitoring, and AE management strategies implemented at their centres, early adopters of tarlatamab can help other institutions develop and refine their own protocols more efficiently. Lessons learned during the early implementation phase, including the roles of various healthcare providers and the transition from inpatient to outpatient care, should facilitate the smoother integration of tarlatamab and other TCEs for solid tumours into clinical pathways across Canada.

## Simple Summary

Small cell lung cancer (SCLC) is an aggressive form of lung cancer that often spreads quickly and is hard to treat after initial therapy. Tarlatamab is a new type of immunotherapy, called a T-cell engager, that helps the body’s immune system attack cancer cells by targeting a protein called DLL3 found on SCLC cells. Clinical trials show that tarlatamab can shrink tumours and help patients live longer, even when other treatments have stopped working. Because these types of drugs can cause serious side effects, hospitals need special plans to monitor patients closely during the first doses. In this paper, Canadian cancer centres are sharing their early experiences to help others adopt this treatment safely and efficiently. The goal is to help guide future care and make these promising therapies more accessible to people across the country.

## 1. Introduction

Lung cancer remains the most diagnosed cancer in Canada, with an estimated 32,000 new cases in 2024 [1]. Small cell lung cancer (SCLC) accounts for approximately 12% of all lung cancer diagnoses [2,3]. This highly aggressive malignancy is characterized by rapid proliferation, early and widespread metastasis, high relapse rates, and substantial morbidity and mortality [4]. Approximately two-thirds of SCLC patients present with metastatic or extensive-stage disease (ES-SCLC) [5]. Although the recent addition of immune checkpoint inhibitors (ICIs) to first-line chemotherapy has improved survival in patients with extensive-stage ES-SCLC, the duration of response and long-term survival remain low [6,7].

In Canada, the recommended first-line systemic therapy for ES-SCLC includes four cycles of etoposide and platinum (EP) in combination with a PD-L1 inhibitor (atezolizumab or durvalumab), followed by PD-L1 maintenance [8]. However, survival gains for ES-SCLC have been modest, with incremental improvements in median overall survival (OS) of approximately 2–3 months when first-line chemotherapy is combined with ICIs versus chemotherapy alone [9]. Subsequent treatment options for ES-SCLC continue to present a significant unmet need and have been the subject of extensive research [10,11,12]. Targeting delta-like ligand 3 (DLL3) is a promising strategy in ES-SCLC because DLL3 is highly expressed on the surface of SCLC tumour cells but largely absent in normal tissues, making it an attractive tumour-specific target [13]. Tarlatamab is a bispecific T-cell engager (TCE) immunotherapy that binds to both DLL3 on cancer cells and the cluster of differentiation 3 (CD3) molecule on T cells, causing T-cell activation, the release of inflammatory cytokines, and the lysis of DLL3-expressing cells [14]. Its efficacy and safety were assessed in the phase 1 DeLLphi-300 (NCT03319940) and phase 2 DeLLphi-301 (NCT05060016) trials and continue to be further evaluated in phase 3 trials and in various clinical settings [15,16]. In DeLLphi-301, after initial step-up dosing, tarlatamab 10 mg every 2 weeks showed antitumour activity with durable responses and promising survival outcomes in previously treated SCLC [16]. Based on the results of these trials, Health Canada approved tarlatamab in September 2024 for the treatment of adult patients with ES-SCLC with disease progression on or after at least two prior lines of therapy, including platinum-based chemotherapy [17]. In March 2025, Canada’s Drug Agency (CDA) issued a positive reimbursement recommendation [18]. In the U.S., the FDA granted accelerated approval to tarlatamab on 16 May 2024, for the treatment of ES-SCLC with disease progression after platinum-based chemotherapy [19]. Recent phase 3 data demonstrated an overall survival benefit for tarlatamab in the second-line setting [12,20], suggesting that this therapy will likely move earlier in the treatment algorithm and become available to an even broader patient population. The demonstrated efficacy and manageable safety profile of tarlatamab solidify its role as a valuable therapeutic agent in previously treated SCLC populations and validate DLL3 as a promising therapeutic target. Building on this success, several other DLL3-targeting strategies (including BI 764532, MK-6070, HPN328) are now being evaluated in both preclinical and clinical studies and are expected to soon expand this therapeutic class [21,22,23]. As tarlatamab is the first TCE approved for ES-SCLC by Health Canada, the learnings described here are based on experiences with this therapy and can be adapted, with modifications if needed, to other similar agents as they become available.

A key challenge with TCEs, including tarlatamab, is the requirement for intensive monitoring during the initial step-up (or ramp-up) dosing, an approach that is relatively new in the management of solid tumours. Although a TCE therapy, tebentafusp, has previously been approved for uveal melanoma, this is a rare malignancy treated almost exclusively at highly specialized centres [24]. In contrast, tarlatamab represents the first TCE approved for a solid tumour with a larger patient population, requiring implementation across both academic and community centres. Furthermore, patients diagnosed with ES-SCLC face unique challenges. Smoking prevalence, highly associated with SCLC, remains higher in lower-income, rural, and marginalized populations in Canada who often have reduced access to specialist care and advanced treatments [25,26,27]. These patients also face longer travel times and higher costs to reach tertiary cancer centres, which can delay curative or palliative interventions. These structural barriers are particularly important for SCLC, because the disease progresses rapidly, and treatment windows are short. Furthermore, due to tobacco exposure, SCLC patients often present with cardiovascular and pulmonary comorbidities that complicate diagnosis and treatment tolerance [28]. Many may already require supplemental oxygen at baseline, either due to these comorbidities or advancing ES-SCLC and are, therefore, at particular risk of severe events even with mild changes in oxygen requirements. In addition, it is estimated that 10–20% of patients with SCLC present with brain metastases at diagnosis, and 50–80% will eventually develop brain metastases during the course of treatment [29], which can present with symptoms that overlap with immune effector cell-associated neurotoxicity syndrome (ICANS).

By offering sustained clinical responses and favourable survival outcomes, tarlatamab addresses a critical treatment gap in previously treated SCLC. However, variations in approaches to cancer care across Canadian centres, primarily driven by available resources and infrastructure (e.g., availability and readiness of inpatient oncology units, day care units, and outpatient clinic setups), can make the adoption of new therapeutic strategies challenging, particularly when a therapy introduces novel administration and monitoring requirements. To date, several Canadian cancer centres, including both specialized cancer centres and community oncology programs, have implemented a collaborative multidisciplinary model of care for the administration of tarlatamab in patients with recurrent ES-SCLC. Their structured approaches, outlined herein, address both the complexity of care required during the tarlatamab initiation phase and the real-world logistical challenges associated with close monitoring and the prompt management of unique, potentially severe, and life-threatening AEs.

## 2. Methodology

This manuscript presents a multicentre expert working group report and a structured narrative review of the early Canadian experience with tarlatamab implementation. Participating centres were purposively selected based on their early adoption of tarlatamab programs in Canada and to ensure representation across different institutional settings and models of care.

To capture cross-Canadian experiences with tarlatamab administration and multidisciplinary collaboration, a questionnaire was distributed to Canadian cancer centres that had initiated tarlatamab treatment programs. The questionnaire addressed both inpatient and outpatient models of care, including the types of healthcare providers involved during regular and after-hours coverage, as well as strategies used to prevent, mitigate, and manage adverse events (AEs).

Following completion of the questionnaires, participating experts met virtually to review and discuss the findings, explore similarities and differences in practice patterns, and share insights from their clinical experience. Participants also contributed relevant documents and clinical algorithms from their respective centres that could be adapted by other institutions planning to implement a tarlatamab program. Draft summaries and proposed pathway components were circulated to all participants for review and feedback to ensure accuracy and consistency with site-specific practices.

The approaches described are intended to provide practical implementation insights and reflect evolving real-world practice. They should not be interpreted as formal clinical guidelines or consensus recommendations but rather as a descriptive overview of early Canadian institutional experience adapted to local resources, workflows, and patient needs.

## 3. Evidence Supporting Tarlatamab in ES-SCLC

DeLLphi-300 evaluated the efficacy of tarlatamab in patients with relapsed/refractory ES-SCLC who received a median of two previous lines of therapy, which demonstrated sustained clinical activity with an overall response rate (ORR) of 25%, a median duration of response (DOR) of 11.2 months, and a median OS of 17.5 months in a heavily pretreated population [15,30].

DeLLphi-301 enrolled a total of 220 patients with ES-SCLC who had previously received ≥2 lines of therapy [16]. Part 1 of the trial (n = 176) was a dose-comparison assessment, after which the 10 mg dose was selected for parts 2 (dose expansion part) and 3 (safety assessment) of the trial. The 10 mg dose provided a more favourable benefit-to-risk profile than the 100 mg dose, with an ORR of 40% (32% in the 100 mg group). Grade ≥ 3 AEs were observed in 59% of patients receiving the 10 mg dose and 64% of those receiving the 100 mg dose, leading to dose modifications in 13% of patients in the 10 mg group and 29% of patients in the 100 mg group. The extended follow-up of DeLLphi-301 demonstrated an ORR and disease control rate (DCR) of 40% and 70%, respectively, with a median progression-free survival (PFS) of 4.3 months and a median OS of 15.2 months [31]. The median OS achieved with tarlatamab in this pretreated patient population is numerically longer than that achieved with chemotherapy + PD-L1 inhibitors in the front-line setting (12.3 months in the IMpower133 [NCT02763579] trial with atezolizumab and 13 months in the CASPIAN trial [NCT03043872] with durvalumab [6,7]).

The phase 3 DeLLphi-304 trial (NCT05740566) is evaluating tarlatamab earlier in the disease course, specifically in the second line (2L) setting. In this study, patients receiving tarlatamab (n = 254) are compared with those receiving standard chemotherapy (topotecan, lurbinectedin, or amrubicin; n = 255). The primary endpoint is OS, with secondary endpoints including ORR, DCR, and duration of DOR. At a median follow-up of 11 months, patients in the tarlatamab arm had significantly longer median OS (13.6 vs. 8.3 months; hazard ratio [HR], 0.60 [95% confidence interval [CI]: 0.47, 0.77]; *p* < 0.001) and median PFS (4.2 vs. 3.2 months; HR, 0.72 [95%CI: 0.59, 0.88]; *p* < 0.001) vs. patients in the chemotherapy arm [12,20]. The occurrence of Grade ≥3 treatment-related adverse events (TRAEs) were lower with tarlatamab vs. chemotherapy (27% vs. 62%), and there were fewer discontinuations due to TRAEs with tarlatamab than chemotherapy (3% vs. 6%). Evolving evidence also suggests that tarlatamab has intracranial activity [32]. In DeLLphi-304, the HR for death demonstrated a higher degree of benefit for tarlatamab compared with chemotherapy in patients with brain metastases (HR = 0.45; 95% CI: 0.31–0.65) [12]. During the study, patients were required to hold tarlatamab if palliative radiation was required. Tarlatamab could be restarted 7 days after the completion of radiation therapy. There are ongoing studies investigating the use of radiation therapy with tarlatamab.

Tarlatamab is also being investigated in the first-line setting, both in combination with induction chemoimmunotherapy and as an addition to maintenance immunotherapy. The phase 1 DeLLphi-303 trial (NCT05361395) reported non-randomized evidence supporting the addition of tarlatamab to maintenance immunotherapy, showing an encouraging 12-month overall survival of 82% and a median OS of 25.3 months from the start of maintenance therapy [33]. In the cohort receiving tarlatamab during induction chemoimmunotherapy (after one cycle of standard treatment), the 12-month OS was 81%, and the median OS was not reached [34].

Results from the ongoing randomized phase 3 DeLLphi-305 trial (NCT06211036), evaluating maintenance tarlatamab plus durvalumab versus durvalumab alone after induction chemoimmunotherapy, are also awaited.

### 3.1. Tarlatamab Safety Profile

The most common AEs (incidence ≥ 20%) in the phase 2 DeLLphi-301 trial in patients treated with a tarlatamab 10 mg dose are listed in Table 1. Other AEs of interest were neutropenia (17%) and ICANS (8%).

Binding of tarlatamab to DLL3 on tumour cells and CD3 on T cells induces immune synapse formation, leading to polyclonal T-cell activation and tumour cell lysis. The resulting rapid cytokine release (including IL-6, interferon-γ, and TNF-α) drives cytokine release syndrome (CRS), which occurs most frequently during early treatment [9,35]. ICANS is believed to result from cytokine-mediated endothelial activation and blood–brain barrier disruption, allowing inflammatory mediators to enter the central nervous system and trigger neuroinflammation [9].

Clearly defined clinical risk factors for CRS with tarlatamab have not been established, as events occur across a broad range of patient and disease characteristics. Suggested contributors from the broader TCE literature include high tumour burden, baseline inflammation, and poor performance status [36,37,38,39] Similarly, specific risk factors for ICANS remain poorly characterized; extrapolated factors include severe or recurrent CRS, elevated inflammatory markers, and pre-existing neurological comorbidities, although the evidence is limited [40,41].

#### CRS and ICANS: Lessons from DeLLphi-301

**Figure 1:**
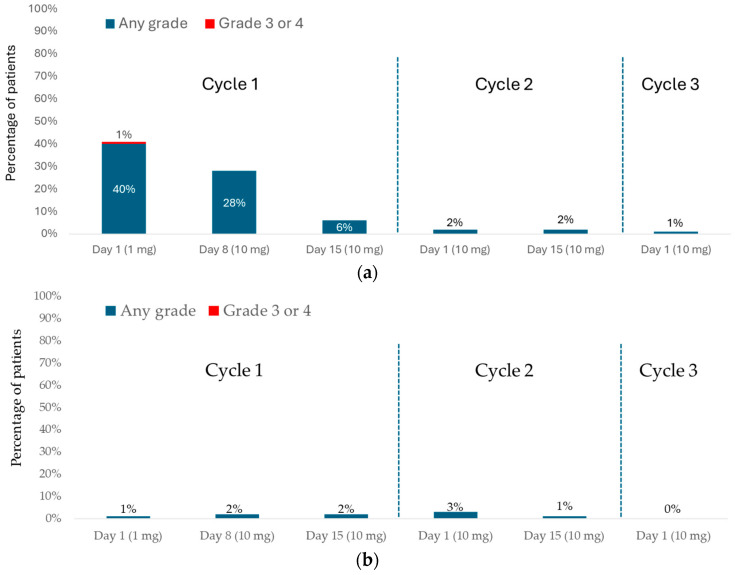
DeLLphi-301: CRS and ICANS during the treatment period. (**a**) CRS; (**b**) ICANS.

In DeLLphi-301, CRS occurred in 68 of 133 (51%) and ICANS in 11 of 133 (8%) patients treated with the 10 mg dose (Figure 1) [16].

CRS most frequently occurred during cycle 1 (C1; 54 of 133 patients [41%] after the first dose (days 1–7), 39 of 133 patients (29%) after the day-8 dose (days 8–14), and 10 of 133 patients [8%] after the day-15 dose (days 15–28). Three of 133 patients (2%) experienced CRS in cycle 2 (C2) or later; all three had missed one or more doses during C1 and resumed treatment in C2. Notably, no patients who completed all three step-up doses in C1 experienced CRS in subsequent cycles. CRS was predominantly Grade 1 (fever only; 40 of 133 patients; 30%) or Grade 2 (fever with hypoxia and/or hypotension not requiring vasopressors; 27 of 133 patients; 20%). Grade 3 CRS was rare, occurring in only one patient (1%) after the 1 mg dose. In total, 24% of patients experienced another CRS event (mostly Grade 1) after receiving a subsequent dose. The most observed CRS symptoms were fever (present in 97% of cases), hypotension (in 20% of cases), and hypoxia (in 16% of cases). CRS typically began approximately 13 h after dosing (median, 13.1 h; interquartile range [IQR], 7.8–27.4 h) and lasted a median of 4 days (IQR, 2–6 days). Most cases were managed with supportive care, including acetaminophen, intravenous fluids, and corticosteroids. More intensive interventions were rarely required (tocilizumab [5% of cases], supplemental oxygen [8%], or vasopressors [1%]). CRS led to dose interruption, dose reduction, or both in 4 of 133 patients (3%), and nearly all cases (98%) resolved.

Most ICANS events and related neurologic symptoms occurred following a tarlatamab dose during C1 (1 of 133 patients [<1%] after the first tarlatamab dose [days 1–7], in 3 of 133 patients [2%] after the day-8 dose [days 8–14], and in 3 of 133 patients [2%] after the day-15 dose [days 15–28], with a median onset after the last tarlatamab dose of 5 days. The median time to onset from the first tarlatamab dose was 30 days (IQR, 13–47 days). All ICANS events were Grade 1 (54%) or Grade 2 (46%).

Symptoms of ICANS can vary in severity. Early manifestations often include headache, reduced attention, impaired handwriting, and mild confusion (for example, difficulty recalling the names of family members). As the condition progresses, more severe symptoms may develop, such as tremor, motor dysfunction, and seizures. In patients receiving the 10 mg dose, ICANS resulted in dose interruption in one patient and treatment discontinuation in another. The median time to resolution of these events was 6.5 days (95% CI, 4.0–17.0).

A similar pattern of CRS incidence, severity, and outcomes was observed in DeLLphi-304 [12], which compared tarlatamab with chemotherapy in the second-line setting. CRS occurred in 56% of patients receiving tarlatamab (142 of 252). Most events were Grade 1 (107 patients) or Grade 2 (32 patients), with only three patients experiencing Grade 3 CRS. CRS occurred mainly during the first two ramp up doses of tarlatamab during C1, and Grade ≥2 events were more frequent after the first dose than after the second. Most cases were resolved with supportive care alone. Glucocorticoids were required in 16% of patients, tocilizumab in 4%, supplemental oxygen in 8%, and vasopressor support in fewer than 1%. ICANS was reported in 6% of patients (15 of 252) in the tarlatamab group. Most events were Grade 1 or Grade 2; however, one fatal (Grade 5) event occurred. This patient’s death was attributed to ICANS in the setting of progressive neurologic decline, accompanied by persistent fever, hypoxemia, and hypotension.

### 3.2. Tarlatamab Dosing and Administration

To reduce the incidence of CRS, tarlatamab is to be administered according to the step-up dosing schedule presented in Table 2 and Table 3 [17].

## 4. Tarlatamab Administration and Canadian Multidisciplinary Models of Care

Before examining the development of models of care for TCEs in solid tumours, it is important to (1) consider the management of SCLC in the Canadian context and (2) review lessons learned from TCEs in hematologic malignancies. These experiences and practice patterns will directly inform the implementation of tarlatamab and guide future integration of other T-cell engagers in solid tumours.

In Canada, larger cancer programs are currently leading the adoption of TCEs and serve as models for smaller institutions. However, many patients with SCLC, who often have a history of tobacco use and come from lower socioeconomic backgrounds, face barriers to accessing care at these centres. Geographic distance, limited transportation, and other travel-related challenges can make treatment far from home difficult. As a result, a substantial proportion of patients with SCLC are managed in smaller community settings, where oncology teams and the infrastructure to initiate TCE therapy may be limited. To promote equitable access, one proposed approach is to administer the first cycle, which includes step-up dosing, at a well-resourced centre, followed by transfer to a local or satellite site for subsequent cycles. This approach would require ensuring that staff at these sites are adequately trained, as such expertise may currently be limited in some jurisdictions. A key challenge with this model arises when treatment is interrupted, as some patients may require re-initiation with step-up dosing. In such situations, clear communication and coordination between primary and satellite centres are essential. For later cycles (C2 and beyond), satellite centres may also rely on provincial guidance to support the safe delivery of treatment. The recommendations and experiences summarized here are intended for centres with the resources and expertise to initiate TCE therapy in patients with SCLC.

The current Canadian experience with TCEs has primarily come from their use in hematologic malignancies, which provides valuable lessons and established protocols that can inform adoption in solid tumours. Some centres with integrated hematology and medical oncology services have leveraged prior experience with T-cell engagers in myeloma and lymphoma, enabling smoother implementation of these therapies for SCLC compared with centres where the two specialties operate separately. Importantly, differences in therapeutic targets influence prophylactic strategies. For example, TCEs in multiple myeloma target B-cell maturation antigen (BCMA) or G protein–coupled receptor, class C, group 5, member D (GPRC5D) and often require antivirals, *Pneumocystis jirovecii* pneumonia (PJP) prophylaxis, and intravenous immunoglobulin (IVIG) to manage hypogammaglobulinemia. In contrast, TCEs in SCLC target DLL3 and generally do not require the same prophylactic measures as those used for TCEs in hematologic malignancies. Prior therapies, particularly those associated with immunosuppression, further influence the discharge prescriptions and infection risk management. Thus, the experience described below focuses on DLL3-directed TCEs in SCLC, with an emphasis on tarlatamab.

The main challenges in establishing models of care for TCEs, including tarlatamab, stem from the complexity of the order sets, which must extend beyond the drug itself to include built-in supportive therapies for AE management, particularly CRS and ICANS. Effective models must also address practical considerations such as after-hours care coverage and the education of multidisciplinary teams.

General steps in establishing TCE delivery programs include staff education, clear definition of inpatient and outpatient team roles and responsibilities, development of treatment and adverse event management protocols, and implementation of comprehensive patient and caregiver education. These key considerations are summarized in Table 4.

### 4.1. Ongoing Targeted Education

#### 4.1.1. Healthcare Providers

Because of their novel mechanism and distinct toxicity profile, TCEs require dedicated and ongoing education for the entire healthcare team. This includes including nursing staff, hospitalists, clinical associates, internal medicine physicians, pharmacists, moonlighters, and on-call providers. Oncology ward teams, who play a central role in monitoring and managing patients throughout the treatment cycle, require focused training on the recognition and management of TCE–associated AEs, particularly CRS and ICANS.

To maintain readiness for an escalation of care in cases of clinical deterioration or severe toxicity, yearly refresher training should also be provided to critical care response teams (CCRT), intensive care unit (ICU) staff, and emergency department personnel. In addition, complex neurotoxicity cases may require a neurologist’s consultation; therefore, neurologists should be familiar with the neurotoxic profile of TCEs and ICANS to support accurate assessment and effective management. A key challenge is ensuring that emergency room (ER) and other staff in peripheral hospitals receive adequate training to recognize and manage these toxicities.

#### 4.1.2. Patients and Caregivers

**Figure A1:**
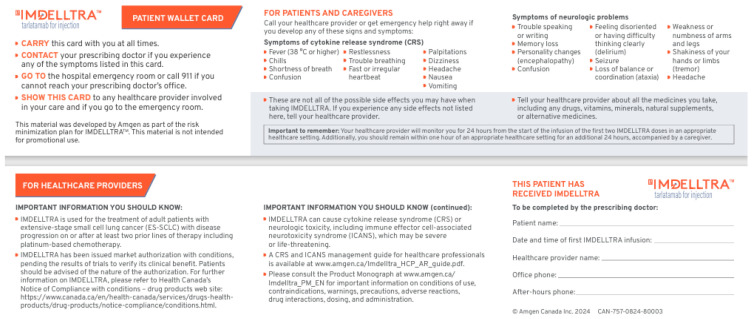
Tarlatamab wallet card.

Patients and caregivers should receive comprehensive education about their treatment and common AEs, with particular emphasis on CRS and ICANS. Education should include the recognition of early warning signs and symptoms and provide clear instructions on whom to contact if these occur. Patients should also receive detailed monitoring guidance, including instructions on when and how to check vital signs, as well as directions for the timely administration of antipyretics or dexamethasone. Maintaining a symptom diary that records the onset, duration, severity, and any interventions is strongly encouraged to support timely communication with the healthcare team and optimize the management of AEs. Patients should be provided with a wallet card to alert healthcare providers that they are receiving a TCE and to highlight the associated risks. Standardized wallet cards are available through Cancer Care Ontario or BC Cancer to ensure consistency and facilitate effective communication in emergency care settings (Appendix A, Table A1). A tarlatamab wallet card (Appendix A, Figure A1) is also available via the patient support program.

### 4.2. Multidisciplinary Care Models: Roles and Responsibilities

**Figure A2:**
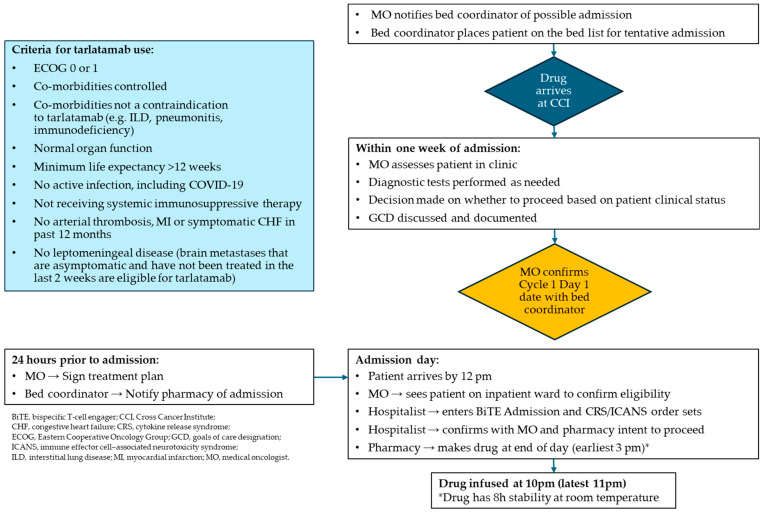
Algorithm for initiation of tarlatamab at Cross Cancer Institute, Edmonton, Alberta.

**Figure A3:**
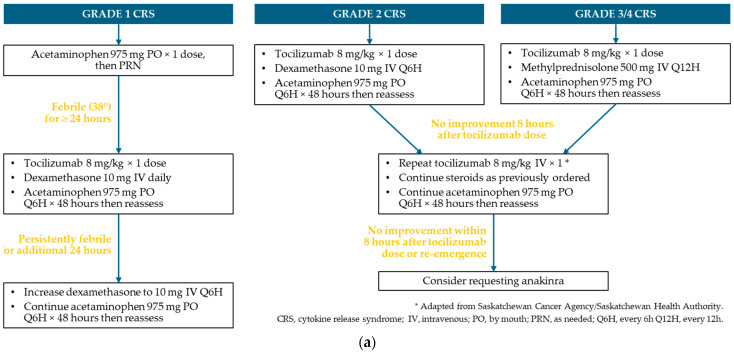

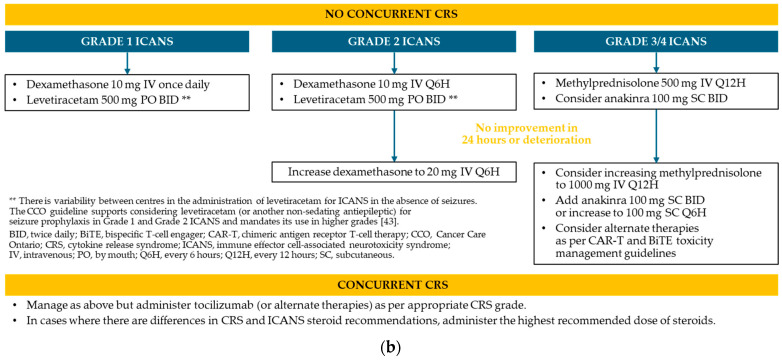
Protocol for the management of CRS and ICANS * at Saskatchewan Cancer Agency. (**a**) CRS; (**b**) ICANS.

**Figure A4:**
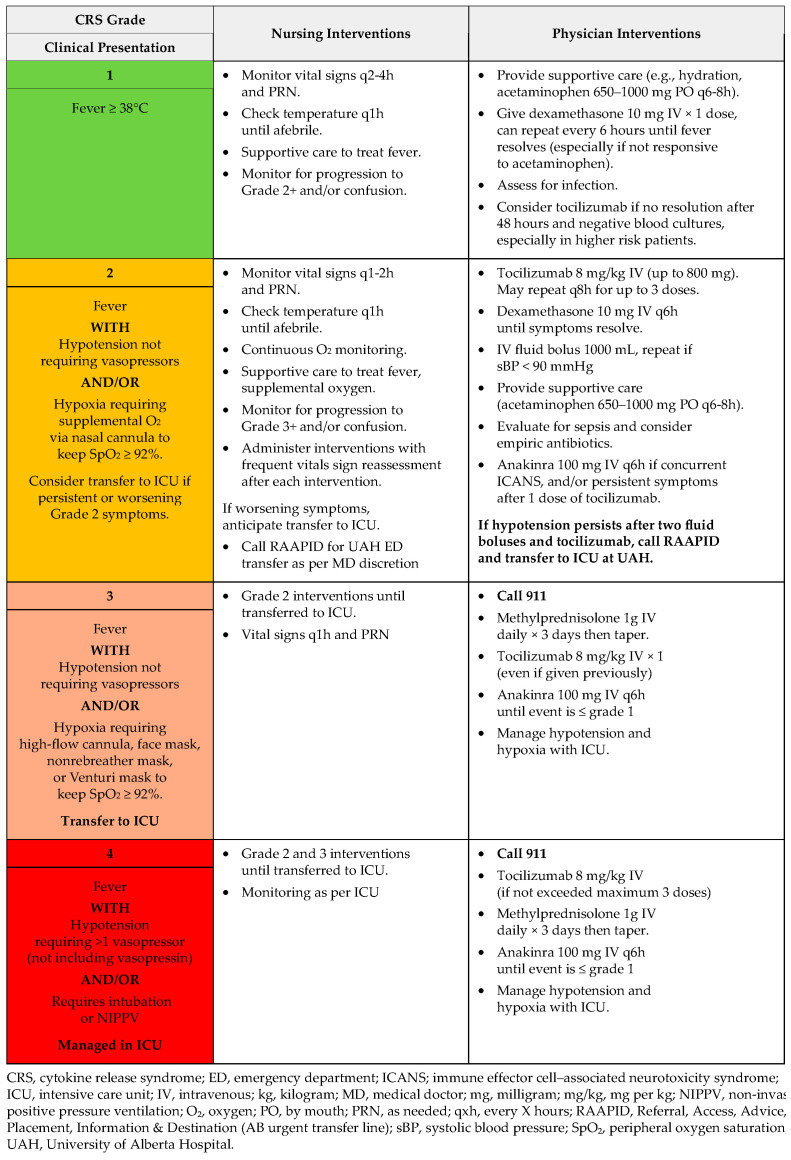
CRS management: T-cell engaging antibodies.

**Figure A5:**
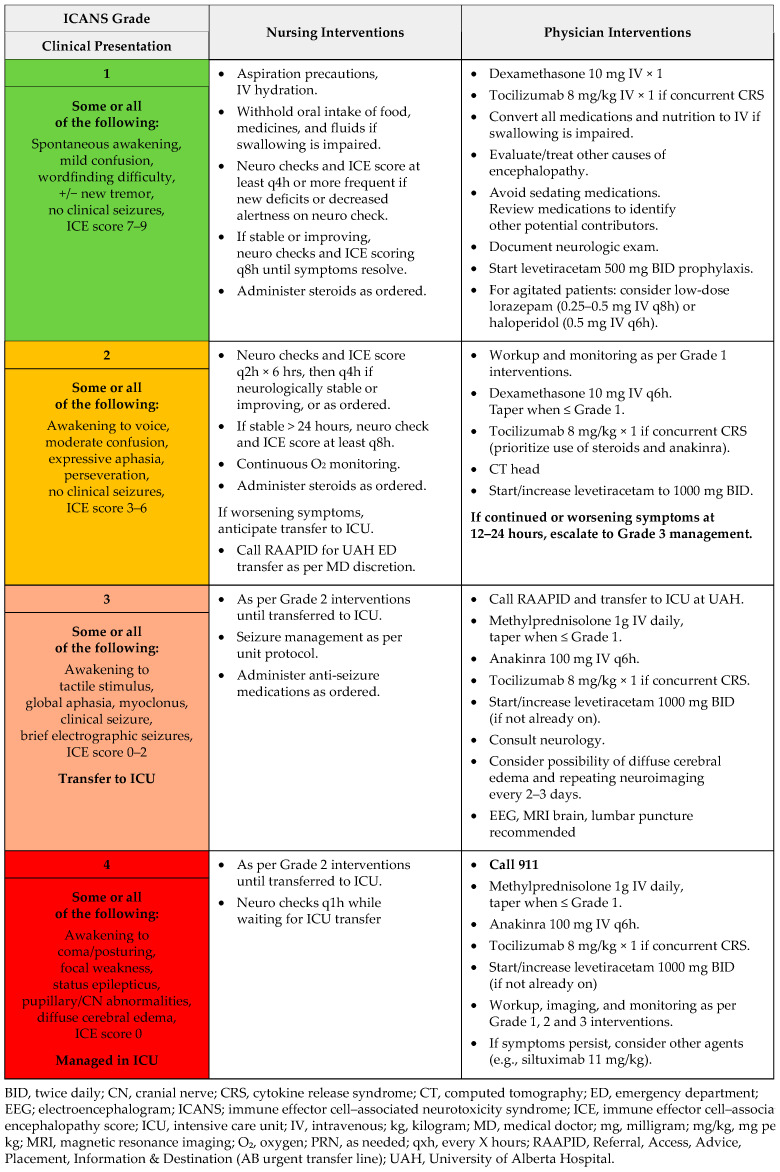
ICANS management: T-cell engaging antibodies.

Although models of care vary between centres (Appendix B, Table A2), several core elements are essential for safe and effective implementation (Table 5). Algorithms and protocols that have been successfully adopted at some cancer centres are provided in Appendix C (Figure A2, Figure A3, Figure A4 and Figure A5) and may be adapted by institutions according to their local infrastructure and available resources.

At Cross Cancer Institute, a dedicated TCE nurse coordinator, experienced with multiple agents beyond tarlatamab, plays a central role in the process (Appendix B, Table A3). The coordinator reviews the treatment plans, educates patients on potential toxicities such as CRS and ICANS, and serves as a liaison with drug access coordinators and the pharmacy to ensure timely drug delivery. Additional responsibilities include organizing inpatient care when required, confirming patient fitness for treatment, monitoring for AEs, and overseeing maintenance needs. This model has been instrumental in reducing inefficiencies and optimizing patient care. A similar approach might be implemented at other centres to enhance coordination, ensure consistent patient monitoring, and optimize the management of TCE therapies.

#### 4.2.1. Oncologist

Once the oncologist determines that a patient is a suitable candidate for tarlatamab, they are responsible for overseeing the subsequent steps in care, ensuring that several key considerations are addressed (Table 5).

Goals of care (GOC) designation and do-not-resuscitate (DNR) status must be discussed and documented in the patient’s chart, with clear notation if ICU admission remains appropriate when required for monitoring or managing reversible treatment-related toxicities. Patients should receive full supportive care, including vasopressors and/or ventilatory support, when clinically indicated and consistent with their goals of care, for the management of reversible toxicities associated with tarlatamab.

Another important step is to ensure the patient can access supportive therapies and that there are no contraindications to supportive care. For example, although central line insertion is not required for all patients, those with difficult venous access may benefit from a peripherally inserted central catheter (PICC) prior to step-up dosing to ensure reliable access if vasopressors are needed. Some centres also perform tuberculosis screening, as this may influence decisions regarding the use of tocilizumab; however, screening practices (such as the Mantoux test or interferon-gamma release assay [IGRA]) vary by region and demographics.

In most centres, TCE order sets include monitoring and management protocols for CRS and ICANS. These should be completed during patient evaluation and preparation, along with documentation of the performance status, comorbidities, and tumour burden, as higher tumour burden can increase the risk of CRS [36,37,38]. In DeLLphi-304, 62% of patients with high tumour burden (sum of longest diameters [SLD] ≥ 72 mm of target lesions) experienced CRS compared with 51% of those with low tumour burden [39]. Although this difference might not appear considerable, patients with high tumour burden are generally more clinically fragile and may be less able to tolerate even mild CRS compared with those with a lower tumour burden.

The oncologist should also consider ordering baseline brain imaging (CT or MRI) prior to treatment, to help differentiate ICANS from brain metastases if neurological symptoms occur. Finally, baseline oxygen requirements should be documented, as patients requiring supplemental oxygen may be at higher risk of CRS-related complications.

#### 4.2.2. Inpatient Care Team

At some centres in Ontario, tarlatamab step-up doses (C1, Days 1, 8, and 15 if needed) are administered in the outpatient clinic, with patients admitted immediately after each dose for monitoring. Some centres in Quebec follow this approach when inpatient wards are at capacity, admitting patients once beds become available later in the morning. These variations highlight the importance of understanding the local workflows and resource constraints at each hospital, centre, or region, and maintaining ongoing communication to optimize patient flow and care delivery.

The most responsible physician (MRP) in the inpatient unit varies by centre, ranging from hospitalists, clinical associates, and general practitioners in oncology (GPOs) to general internal medicine physicians or oncologists. Care is delivered by a multidisciplinary team to address both medical and supportive needs. Inpatient management typically involves the attending oncologist, either as MRP or consultant, supported by nurses, nurse practitioners, fellows, clinical associates, and/or hospitalists (Appendix B, Table A2).

After-hours coverage varies, with oncology fellows at academic centres or on-call in-ternal medicine/oncology physicians providing this support. The CCRT/ICU and neurology teams should be alerted for patients at risk of CRS or ICANS to ensure timely intervention. Since not all centres have a dedicated CCRT team, each care model should clearly define first-call responsibilities for patients exhibiting symptoms that may require ICU-level care.

#### 4.2.3. Outpatient Management

The transition from inpatient to outpatient care is influenced by several factors, including patient tolerability during C1, absence of high-grade toxicities, availability of adequate outpatient monitoring resources, and the reliability of the patient’s social support.

For outpatient management, several centres provide dedicated TCE support during business hours, while others rely on specialized nurses or nurse practitioners. Some centres also have outpatient daycare units or supportive care clinics where patients can be assessed if they develop signs or symptoms of CRS; however, these units typically close around 4–5 pm. After hours, patients are instructed to present to the nearest ER. Patients are generally advised to contact their oncology team before presenting to the ER, allowing physicians to brief the ER staff in advance. This highlights the need for targeted education of ER staff. Patients are also provided with wallet cards and instructed to present these cards to ER personnel to ensure timely and appropriate care.

## 5. Practical Considerations for Tarlatamab Implementation and Delivery

**Figure 2:**
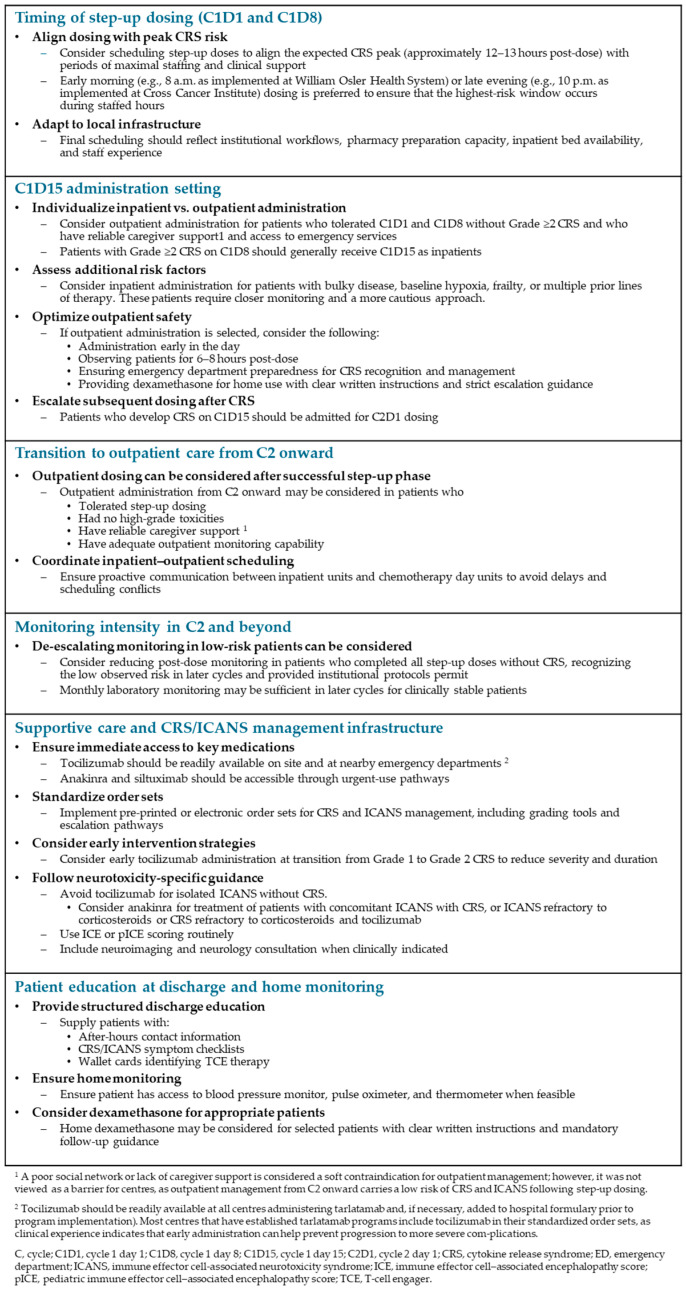
Practical considerations for tarlatamab implementation and delivery.

Figure 2 summarizes practical considerations for tarlatamab implementation and delivery derived from Canadian expert experience. Because these recommendations are based on individual institutional practices and clinical judgment, healthcare providers should also refer to the product monograph.

Clinical trials evaluating outpatient step-up dosing are ongoing [42], and administration requirements may evolve as more data become available. In practice, scheduling can also be challenging, particularly in outpatient settings, where infusion slots may compete with other treatments. Adequate communication between the ward and chemotherapy daycare is essential to ensure proper coordination and continuity in treatment planning.

### 5.1. Supportive Care and Management of CRS and ICANS

To ensure that all healthcare providers involved in care, including emergency department staff, are aware of the potential risks and can respond promptly, some centres have included information about CRS and ICANS in the tarlatamab order sets and/or patient charts (Appendix B, Table A4). Appendix C (Figure A4 and Figure A5) provides details on CRS/ICANS grading and outlines the roles of nurses and physicians in their management. It is important to remember that CRS is defined as a systemic inflammatory response that can lead to dysfunction in any organ.

For ICANS, order sets often include brain imaging in consultation with neurology. Some centres perform CT scans for Grade 1–2 ICANS and reserve MRI for follow-up as needed. The Immune Effector Cell–Associated Encephalopathy (ICE) score is routinely used by nursing staff during admission to monitor for neurotoxicity. Assessing a patient’s writing ability is a practical and reliable way to detect changes associated with ICANS; however, literacy or language barriers may require relying on caregiver observations. In such cases, the pediatric ICE (pICE) score may be used and has been adopted by some centres.

#### 5.1.1. Practical Considerations for Dexamethasone at Discharge

In certain situations, CRS treatment dose of dexamethasone (8 mg) may be prescribed as a take-home medication. This approach is simple, cost-effective, and enables early symptom management, particularly for patients who reside far from care centres or may encounter lengthy emergency room wait times. Patients should receive explicit instructions to call their healthcare team if symptoms develop, take dexamethasone only under the direction of their healthcare provider, and seek immediate medical assessment afterward. While having dexamethasone on hand can be helpful, there is a potential downside: if a patient feels better by the time they reach the ER, they may be sent home. This underscores the importance of clear education for patients, caregivers, and ER staff, as well as prompt follow-up.

#### 5.1.2. Tocilizumab and Anakinra Use in Routine Practice

Although DeLLphi-301 reported low use of tocilizumab (in 5% of CRS cases), the median duration of CRS was prolonged, approximately 4 days (IQR, 2–6 days). In contrast, all but one Canadian centre participating in this initiative administers tocilizumab early to help reduce the duration and severity of CRS events. Tocilizumab is typically considered at the transition from Grade 1 to Grade 2 CRS, as earlier intervention can mitigate symptom escalation and facilitate earlier discharge.

Tocilizumab is typically pre-ordered and put on hold for patients prescribed TCEs, ensuring immediate availability if escalation is needed. Physicians should also confirm that satellite hospitals or nearby emergency departments have tocilizumab available. The infusion takes approximately one hour and can be repeated up to three times within 24 h (every eight hours, maximum of four doses), with monitoring for potential liver enzyme elevations [43]. In some centres, if additional intervention is required after two doses, treatment is switched to anakinra, a recombinant IL-1 receptor antagonist, which has proven effective for managing refractory CRS and is generally available [44,45]. In addition, as per Cancer Care Ontario (CCO) guidelines, tocilizumab is not recommended for ICANS in the absence of concurrent CRS, as it does not achieve meaningful drug levels in the cerebrospinal fluid and could theoretically worsen ICANS [46,47]. For patients with concurrent CRS, there is a low threshold to switch to anakinra. Anakinra doses vary in the literature and are based on the clinical scenario. Doses up to 400 mg total per day have been reported, and a minimum of 100 mg per day is recommended. CCO recommends that physicians reach out to centres with more experience for advice if needed. The Saskatchewan Cancer Agency has preprinted order sets for anakinra. The current dosing is 100 mg subcutaneously every 12 h for patients >50 kg and 2 mg/kg every 12 h for those <50 kg. If there is no improvement on this schedule, the order set includes a note to consider increasing the frequency to every 6 h. The preprinted order set also clearly states that anakinra should be considered after two doses of tocilizumab when treating CRS.

Although evidence supporting the prophylactic use of tocilizumab with TCEs in solid tumours is limited, experience with teclistamab, a bispecific TCE approved for relapsed/refractory multiple myeloma, demonstrated that prophylactic tocilizumab can reduce the incidence and severity of CRS, with no hospital readmissions within 14 days of discharge [48]. Data on prophylactic tocilizumab use in patients treated with tarlatamab are still evolving, and its use should be guided by clinical judgment. In select cases, oncologists may consider prophylactic tocilizumab for medically fragile patients, such as those requiring supplemental oxygen (where even mild hypoxemia could have serious consequences) or those with heart failure or severe renal impairment, in whom avoiding CRS-related hypotension and subsequent fluid overload from intravenous fluid administration is particularly important.

#### 5.1.3. Management of Other Tarlatamab AEs

It is speculated that taste bud cells express DLL3 and that tarlatamab may lead to T–cell-mediated destruction of these DLL3-expressing taste bud cells, causing dysgeusia [49]. In DeLLphi-301, dysgeusia was reported in 42 of 133 patients (32%), with a median time to onset of 34 days (IQR, 21–51 days) [16]. In DeLLphi-304, 58 of 252 patients (23%) experienced dysgeusia (43% Grade 1 and 15% Grade 2). There was no treatment discontinuation due to dysgeusia [39].

Management strategies include the use of ice chips during and after treatment or cold beverages to alleviate symptoms (theoretically by reducing absorption through the tongue), maintaining good oral hygiene, periodically chewing cardamom, and inhaling the scents of cloves and lemon [50]. Patients can also be encouraged to explore foods with different, intense, and complex flavors. Zinc supplements can be recommended to help improve dysgeusia by supporting taste bud function and regeneration [51]. Patients may experience flu-like symptoms and fatigue, particularly during the first month of treatment. These symptoms can be distressing and may lead to treatment discontinuation if not properly managed. Transient elevations in liver function tests (LFTs) have also been observed; however, these need to be distinguished from elevations related to tocilizumab. Providing patients with information that these effects are usually temporary and will subside can help alleviate anxiety and encourage treatment continuation.

Patients with a high tumour burden may experience significant pain [52]. Ensuring the availability of appropriate analgesics, including opioids, is crucial for effective pain management. Tailoring pain management strategies to individual patient needs can enhance comfort and improve overall treatment outcomes.

### 5.2. C2 and Beyond: Monitoring

The administration of complex therapies in a resource-constrained healthcare system requires healthcare providers to identify strategies for simplifying and streamlining outpatient management. Clinical experience suggests that the intensive post-dose monitoring recommended in the tarlatamab product monograph may not always be necessary, particularly for patients who have tolerated the step-up dosing without complications. Available data also indicate that the risk of CRS decreases after C2, especially in patients with no prior history of CRS. In DeLLphi-301, no patients who completed all three step-up doses in C1 experienced CRS in C2 or later. Notably, patients who developed CRS in C2 or later had missed one or more C1 doses and resumed treatment in C2.

While some centres continue to follow the product monograph closely for Cycles 2 and 3, accumulating experience has allowed others to shorten the monitoring periods, given the minimal risk of CRS and ICANS in C2 and beyond. From Cycle 3 onward, some centres have reduced post-dose monitoring in the systemic therapy suite to as little as one hour. Nonetheless, caution is advised until more definitive evidence supports this approach. In later cycles, frequent blood work may also be unnecessary, with many centres transitioning to once-per-month testing.

## 6. Future Directions and Adaptation

While current models of care prioritize patient safety and adherence to treatment protocols, several logistical challenges remain, including limited outpatient monitoring capacity and the need for seamless coordination between inpatient and outpatient teams. Canadian centres are actively working to expand outpatient capabilities for tarlatamab C1D15 monitoring and subsequent doses beyond C1. This evolving model reflects a focus on safety, collaboration, and continuity, with ongoing efforts to streamline outpatient delivery, enhance patient experience, improve resource efficiency, and implement nursing-led protocols for triaging low-grade CRS or ICANS that may not require hospitalization.

Ongoing phase 3 trials, DeLLphi-304 (second-line SCLC), DeLLphi-305 (first-line maintenance for extensive-stage SCLC), and DeLLphi-306 (NCT06117774; consolidation in limited-stage SCLC), are evaluating strategies to shorten outpatient monitoring after tarlatamab dosing and enhance home- and clinic-based oversight. The integration of remote patient monitoring with wearable devices can enable early detection of fever, hypoxia, or hypotension, facilitating timely triage and intervention.

This work reflects early real-world experience from a purposively selected group of Canadian centres with established tarlatamab programs and may therefore overrepresent practices from highly specialized institutions with larger infrastructure and multidisciplinary support. No systematic or comparative evaluation of clinical outcomes across different models of care was performed, and the approaches described are based on observational experience and expert opinion. As such, some workflows may not be directly transferable to smaller or less-resourced settings without local adaptation. Nevertheless, this report provides a practical resource for centres seeking to develop or refine their own implementation pathways, offering transferable principles and real-world insights that can be adapted to diverse clinical environments.

Although this work is grounded in the Canadian healthcare context, several of the operational elements described are broadly applicable to other healthcare systems. System-specific factors include provincial drug funding mechanisms, regional referral pathways, availability of specialized oncology units, and the use of satellite centres coordinated through provincial cancer agencies. In contrast, the core principles underpinning successful TCE implementation, early multidisciplinary engagement, standardized CRS/ICANS protocols, step-up dosing pathways, clear inpatient–outpatient transitions, predefined eligibility criteria, caregiver education, and rapid access to immunomodulatory therapies, are generalizable across jurisdictions. These foundational components may be adapted to diverse reimbursement models and institutional structures, providing a practical framework for centres internationally as TCEs expand into solid tumours.

## 7. Conclusions

The administration of tarlatamab, along with the various models of care described here, demonstrates the importance of proactive coordinated approaches for integrating novel therapies into clinical practice. There is no one-size-fits-all approach to implementing tarlatamab at a centre. The approach depends on factors such as the inpatient structure (dedicated oncology unit vs. general medicine admission), the on-call oncology service, the willingness of internal medicine teams to participate in patient care, the availability of CCRT, and the access to specialized oncology healthcare providers (nurse practitioners, fellows, residents, physician assistants, etc.). Despite these variations, tarlatamab has been safely administered across numerous Canadian institutions. By leveraging interdisciplinary teamwork, standardized protocols, and patient-centred planning, cancer centres can deliver this innovative treatment safely while addressing logistical challenges such as outpatient monitoring, caregiver support, and transitions between care settings.

As healthcare systems continue to adapt to new classes of oncology agents, experiences and models like these will be crucial for maintaining high standards of care, improving patient experience, and optimizing outcomes in the era of precision immunotherapy. Continued evaluation, real-world learning, and refinement of workflows will ensure that these approaches remain scalable, efficient, and responsive to the evolving landscape of TCE therapies. While specific implementation pathways will vary by healthcare system, the clinical and operational principles outlined in this paper are broadly transferable and may inform global adoption of tarlatamab and other TCEs in solid tumours.

## Figures and Tables

**Table 1:** Incidence of common AEs (≥20%) with 10 mg tarlatamab in DeLLphi-301 [16].

AEs	Tarlatamab 10 mg (Parts 1 + 2 + 3; N = 133) n (%)
Patients with AEs	130 (97.7)
Cytokine release syndrome (CRS)	68 (51.1)
Decreased appetite	38 (28.6)
Pyrexia	46 (34.6) ^1^
Constipation	36 (27.1)
Anemia	35 (26.3)
Asthenia	30 (22.6)
Dysgeusia	38 (28.6)
Fatigue	30 (22.6)

^1^ The pyrexia events reported were not identified as CRS as assessed by the investigators.

**Table 2:** Product monograph recommended dosage schedule of tarlatamab [17].

Dosing Schedule	Days	Tarlatamab Dose	Recommended Monitoring
Cycle 1	1 ^a^	1 mg ^a^	Monitor patients from the start of the tarlatamab infusion for 24 h on Cycle 1 Day 1 and Cycle 1 Day 8 in an appropriate healthcare setting
	8 ^a^	10 mg ^a^	Recommend that patients remain within 1 h of an appropriate healthcare setting for a total of 48 h from the start of the infusion with tarlatamab, accompanied by a caregiver
	15	10 mg	Observe patients for 6–8 h post tarlatamab infusion ^b^
Cycle 2	1, 15	10 mg	Observe patients for 6–8 h post tarlatamab infusion ^b^
Cycles 3–4	1, 15	10 mg	Observe patients for 3–4 h post tarlatamab infusion ^b^
Cycle 5 and subsequent infusions	1, 15	10 mg	Observe patients for 2 h post tarlatamab infusion ^b^

^a^ Administer recommended concomitant medications before and after Cycle 1 infusions; ^b^ Extended monitoring in a healthcare setting is not required unless the patient experiences Grade ≥ 2 CRS, ICANS, or neurological toxicity during prior treatments or physician assessment of individual patient risk.

**Table 3:** Product monograph recommended concomitant medications for tarlatamab administration for cycle 1 [17].

Days	Medication	Administration
1, 8	8 mg of dexamethasone intravenously (or equivalent)	Within 1 h prior to tarlatamab administration
1, 8, 15	1 L of normal saline intravenously over 4–5 h	Immediately after completion of tarlatamab infusion

**Table 4:** Setting up delivery of tarlatamab: steps to consider prior to starting your program.

Staff education	Ensure adequate training and education for all HCPs involved in care of patients treated with TCEs—including but not limited to inpatient and outpatient general GPOs, hospitalists that act as MRP, internal medicine teams that may evaluate the patient in the ER or the ward, nursing, pharmacists, and ER, CCRT, and ICU staff
	Educate neurology teams regarding ICANS management
Inpatient care team structure	Define which HCPs are the first call for patients during the day, evening, weekends, and after hours
Preprinted protocols and medical directives	Prepare standardized inpatient order sets with clear medical directives to ensure nurses can promptly administer tocilizumab and dexamethasone or methylprednisolone when needed
Patient and caregiver education	Ensure sufficient patient/caregiver education materials are in place
	Ensure patients have documents (e.g., wallet card) to present to the HCP in an urgent care facility, to allow prompt recognition of TCE toxicity and management
Tarlatamab dosing schedule	Determine the timing of tarlatamab administration prior to program initiation to ensure monitoring requirements are met while maximizing the availability of experienced staff on site
Medication/imaging accessibility	Ensure tocilizumab is readily available on the unit for rapid administration
	Ensure tocilizumab is readily available at neighboring centres where patients may present to ER
	Ensure rapid access to anakinra for patients with concomitant ICANS with CRS, ICANS refractory to corticosteroids, or CRS refractory to corticosteroids and tocilizumab ^1^
	Ensure access to CNS imaging for patients with suspected ICANS

^1^ In some institutions, initiation of T-cell engager programs requires tocilizumab and anakinra to be added to the hospital formulary in advance to ensure timely access for the management of CRS and ICANS. CCRT, critical care response teams; CNS, central nervous system; CRS, cytokine release syndrome; ER, emergency room; GPO, general practitioner in oncology; HCP, healthcare provider; ICANS, immune effector cell-associated neurotoxicity syndrome; ICU, intensive care unit; MRP, most responsible physician, TCE, T-cell engager.

**Table 5:** Model of care for the safe delivery of tarlatamab: key elements.

Patient Evaluation and Preparation	C1: Inpatient Care	C2 and Beyond: Outpatient Care
Oncologist	Tarlatamab Dosing (IV Over 60 min) Supportive Care Medications	
Confirm eligibility criteria for tarlatamab are met. Review and document performance status, comorbidities, and tumour burden. Document any abnormalities in baseline vital signs. Document if on supplemental oxygen at baseline. Consider baseline brain imaging (CT or MRI). Assess venous access needs. Review TB history if relevant; consider TB testing with either a skin test or QuantiFERON assay. Document goals of care. Distinguish between reversible and irreversible causes when determining the need for life-supportive measures. Complete standardized admission orders for tarlatamab and management orders for CRS and ICANS.	D1 and D8: → 8 mg dexamethasone; IV one hour prior to tarlatamab D1, D8, and D15: → 0.9% NaCl 1 L; IV (200–250 mL/hour) post-tarlatamab	Concomitant supportive care medications for C2 and beyond are not required.
	C1 inpatient monitoring ^1^ and discharge	Discharge to outpatient setting
	C1, D1: 24 h → Discharge, if stable ^2^ Inpatient or outpatient care team arranges for D8 administration C1, D8: 24 h → Discharge, if stable ^2^ Inpatient or outpatient care team arranges for C1, D15 administration (inpatient or outpatient) C1, D15 (if admission required): 24 h → Discharge, if stable ^2^ Inpatient or outpatient care arranged for C2, D1 administration.	The inpatient team coordinates the transition to the outpatient setting. C1, D15 ^3^ and/or C2, D1 ^3^

^1^ Vitals (T, RR, SpO_2_, BP, HR), CRS, and ICANS screening. Frequency of monitoring: Assess prior to each tarlatamab dose and every 4 h afterward. CRS monitoring should increase based on severity (e.g., every 2 h at the onset of Grade 2 CRS). ICE scores should be assessed every 12 h, with increased frequency at the onset of ICANS symptoms (e.g., every 8 h for Grade 1 ICANS). ^2^ 24–48 h max depending on AEs and patient’s overall status. ^3^ Follow institution/centre protocol for post-dose monitoring requirements. AE, adverse event; BP, blood pressure; C, cycle; CRS, cytokine release syndrome; CT, computed tomography; D, day; HR, heart rate; ICANS, immune effector cell-associated neurotoxicity syndrome; IV, intravenous; MRI, magnetic resonance imaging; NaCl, sodium chloride; RR, respiratory rate; T, temperature; TB, tuberculosis; SpO_2_, oxygen saturation.

## Data Availability

No new data were created or analyzed in this study.

## Abbreviations

The following abbreviations are used in this manuscript:

AEs	Adverse events
BCMA	B-cell maturation antigen
BP	Blood pressure
C	Cycle
CCRT	Critical care response team
CD3	Cluster of differentiation 3
CDA	Canada’s Drug Agency
CI	Confidence interval
CNS	Central nervous system
CRS	Cytokine release syndrome
CT	Computed tomography
D	Day
DCR	Disease control rate
DLL3	Delta-like ligand 3
DOR	Duration of response
EP	Etoposide and platinum chemotherapy
ER	Emergency room
ES-SCLC	Extensive-stage small cell lung cancer
FDA	U.S. Food and Drug Administration
GPO	General practitioner in oncology
GPRC5D	G protein-coupled receptor class C group 5
HCP	Healthcare provider
HR	Hazard ratio
ICANS	Immune effector cell-associated neurotoxicity syndrome
ICEscore	Immune Effector Cell-Associated Encephalopathy score
ICI	Immune checkpoint inhibitor
IGRA	Interferon-gamma release assay
IQR	Interquartile range
IV	Intravenous
IVIG	Intravenous immunoglobulin
MRI	Magnetic resonance imaging
MRP	Most responsible physician
ORR	Overall response rate
PD-L1	Programmed death-ligand 1
PFS	Progression-free survival
PICC	Peripherally inserted central catheter
pICE	Pediatric Immune Effector Cell-Associated Encephalopathy score
PJP	Pneumocystis jirovecii pneumonia
RR	Respiratory rate
SCLC	Small cell lung cancer
SpO_2_	Oxygen saturation
TB	Tuberculosis
TCE	T-cell engager

## Appendix A

**Table A1:** Hyperlinks to provincial educational materials.

Province	Available Educational Materials
Ontario	Treating cancer with T-cell engaging antibodies: What you need to know
Alberta	Cancer Care Alberta: Information for patients/families bispecific antibodies
British Columbia	SCCRS & SCICANS patient handout Bispecific antibodies patient letter Bispecific antibodies alert card Bispecific antibodies nursing resources
Nova Scotia	Cell Therapy and Transplant Program

## Appendix B

**Table A2:** Model of care for the safe delivery of tarlatamab: provider responsible for monitoring and/or to attend patient calls.

Centre	Attending Provider		DAY 15 (Tarlatamab Admin)	OP (During Hours)		OP (After Hours)	
	IP (During Hours)	IP (After Hours)		Assessment If Signs/Symptoms of CRS/ICANS	On-Call Provider	Assessment If Signs/Symptoms of CRS/ICANS	On-Call Provider
QEII Health Sciences Centre	Clinical associates and MO	In-house IM residents	IP	Supportive care clinic or ER	Primary MO	ER	MO
Saint John Regional Hospital	Attending MO or clinical associate	On call MO	OP facility	OP clinic or phone triage	Nurse triages to MO	ER	MO
Hôpital du Sacré-Cœur- de-Montréal	On call attending physician		OP facility	ER	Treating physician or physician	ER	Physician
Hôpital Charles- Le Moyne	On call staff		IP	OP clinic	Pivot nurse and treating clinician	ER	Nurse (for symptoms), then MO
Princess Margaret Cancer Centre	On call fellow		OP facility	OP clinic or ER	Staff	ER *	Staff
William Osler Health System	Oncology hospitalist or GPO	General IM	IP (working towards OP)	OP oncology care urgent clinic or ER	Nurse triages to MO	ER *	ER physician, MO
Stronach Regional Cancer Centre	Nursing and MO supported by NP		OP facility	OP clinic	Nurse triages to MO	ER *	ER physician
London Health Sciences Centre	GP hospitalists	Residents or fellows or IM medicine moonlighters with staff oncologist support	OP facility	Solid tumour assessment clinic	Patient’s primary oncologist, with TCE champion support	ER *	MO
Saskatoon Cancer Centre	Clinical associate and MRP	On call MO	IP or OP facility	ER	Oncologist through nursing intake	ER	MO
Cross Cancer Institute	Hospitalists and MRP	Hospitalists and on-call physician (with phone support of MRP)	OP facility	IP	MRP or covering physician	ER	Physician
BC Cancer Vancouver	On call fellow		OP facility	OP clinic or ER	Staff	ER	Staff

* In Ontario, patients can contact CareChart Oncology, an after-hours virtual support program for patients undergoing cancer treatment, sponsored by Ontario Health (Cancer Care Ontario). Admin, administration; BC, British Columbia; ER, emergency room; GPO, general practitioner in oncology; IM, internal medicine; IP, inpatient; MO, medical oncologist; MRP, most responsible physician; NP, nurse practitioner; OP, outpatient; QEII, Queen Elizabeth II; SpO_2_, oxygen saturation; TCE, T-cell engager.

**Table A3:** Summary of T-cell engager nurse coordinator workflow at Cross Cancer Institute ^1^.

Task	Key Steps/Actions
Referral and case review	Review diagnosis, treatment plan, prior therapies, and potential barriers (e.g., drug coverage, lodging)
First patient contact	Discuss regimen and potential side effects (CRS, ICANS, skin, infections); review admission expectations; provide patient education materials; confirm start date
Coordination	Liaise with DAC, SAP pharmacy, and PSP for funding approval and medication arrival
Organize inpatient admission	Notify bed coordinator; add hematology admits to BMT rounds; inform CAR-T physician
Pre-admission	Confirm labs and clinic visits; ensure goals of care; apply treatment plan within 24 h; ensure pre-medications as needed
During admission	Monitor step-up dosing; track and manage adverse reactions
Discharge	Coordinate follow-up appointments; document infusion clinic information; provide ER letter and emergency contact card; educate on side effect management
Post-admission follow-up	Monitor infection prophylaxis; notify provider if doses missed
Repeat step-up dosing	Organize re-admission if delayed; provider requests assistance via secure chat

^1^ Includes both bispecific antibodies and CAR-T cell therapy. BMT, bone marrow transplant; CAR-T, chimeric antigen receptor T-cell therapy; CRS, cytokine release syndrome; DAC, drug access coordinator; ICANS, immune effector cell-associated neurotoxicity syndrome; PSP, patient support program; SAP, special access programme.

**Table A4:** CRS and ICANS in tarlatamab order set at Saskatchewan Cancer Agency.

Tarlatamab Cycle 1 Order Set Indication: SCLC			
Protocol			
Tarlatamab 1 mg		Day 1	
Tarlatamab 10 mg		Day 8, 15	
Mandatory admission	Patient must be admitted until 24 h after administration of the Cycle 1 Day 8 dose of 10 mg.		
CRS incidence All grades (Grades 3–4)	55% (1.6%)	Median onset: 13.5 h (1–268 h) Median duration: 4 days (2–6 days) Most likely to occur after the first two doses	
ICANS incidence All grades (Grades 3–4)	9% (0%)	Median onset: 29.5 days Median duration: 33 days (1–93 days)	
Tocilizumab use	5–10%		

CRS, cytokine release syndrome; ICANS, immune effector cell-associated neurotoxicity syndrome; SCLC, small cell lung cancer.
